# Ferroptosis‐related gene *NOX4*, *CHAC1* and *HIF1A* are valid biomarkers for stomach adenocarcinoma

**DOI:** 10.1111/jcmm.17171

**Published:** 2022-01-13

**Authors:** Ruoxi Xiao, Shasha Wang, Jing Guo, Shihai Liu, Aiping Ding, Gongjun Wang, Wenqian Li, Yuqi Zhang, Xiaoqian Bian, Shufen Zhao, Wensheng Qiu

**Affiliations:** ^1^ Department of Medcine Qingdao University Qingdao China; ^2^ Department of Oncology Affiliated Hospital of Qingdao University Qingdao China

**Keywords:** *CHAC1*, ferroptosis, *HIF1A*, *NOX4*, prognostic signature, stomach adenocarcinoma

## Abstract

Ferroptosis is a regulated cell death nexus linking metabolism, redox biology and diseases including cancer. The aim of the present study was to identify a ferroptosis‐related gene prognostic signature for stomach adenocarcinoma (STAD) by systematic analysis of transcriptional profiles from The Cancer Genome Atlas (TCGA), GEO and a clinical cohort from our centre. We developed a predictive model based on three ferroptosis‐related genes (*CHAC1, NOX4* *and* *HIF1A*), gene expression data and corresponding clinical outcomes were obtained from the TCGA database, and the reliability of this model was verified with GSE15459 and 51 queues in our centre. ROC curve showed better predictive ability using the risk score. Immune cell enrichment analysis demonstrated that the types of immune cells and their expression levels in the high‐risk group were significantly different from those in the low‐risk group. The experimental results confirmed that *NOX4* was upregulated and *CHAC1* was downregulated in the STAD tissues compared with the normal stomach mucosal tissues (*p* < 0.05). In sum, the ferroptosis‐related gene signature can accurately predict the outcomes of patients with STAD, providing valuable insights for personalized treatment. As the signature also has relevance to the immune characteristics, it may help improve the efficacy of personalized immunotherapy.

## 1 INTRODUCTION

Stomach cancer is one of the most common malignancies worldwide and is the third leading cause of cancer‐related death. It is a major unmet clinical problem with more than 1 million new cases around the world each year.^1^ Stomach cancer is a multifactorial disease and a variety of pathogenic infections contribute to its occurrence and development, such as *Helicobacter pylori* and Epstein Barr virus. A major fraction of stomach cancer can be efficiently prevented by applying *H*. *pylori* eradication therapy. Today, the 5‐year survival for stage IA and IB tumours treated with surgery is between 60% and 80%. However, the low rate of early diagnosis means that most patients have advanced‐stage disease at diagnosis, so the best surgical window is missed. Therefore, the standard treatment for advanced stomach cancer combines neoadjuvant chemoradiotherapy, molecular‐targeted therapy and immunotherapy. Moreover, the dramatic development of immune checkpoint inhibitors, such as *CTLA‐4* and *PD‐1*, suggests amazing therapeutic effects in clinical efficacy. However, therapy conditions are not eligible for most STAD patients, which suggests that more studies on the molecular mechanisms’ elucidation and identifying useful biomarkers for immune checkpoint inhibitors are still urgently needed for cancer immunotherapy.

The Stockwell BR laboratory first proposed the concept of ferroptosis in 2012.^2^ Ferroptosis is a novel form of programmed cell death that is distinct from apoptosis, necroptosis and autophagy in terms of its genetics, cell morphology and biochemical function.^3^ This process is significantly distinctive because of the catastrophic accumulation of reactive oxygen species (ROS) and abnormal iron metabolism. An initial characterization of the mechanism triggering ferroptosis is cysteine depletion, which leads to the exhaustion of glutathione (GSH) intracellularly.^4^ Hence, it is conceivable that a complex interplay that regulates the different cancer cell susceptibilities to ferroptosis would be a fruitful area in cancer research. Many studies have confirmed that many genes are involved in the initiation and execution of ferroptosis in cancers.^5^, ^6^, ^7^

By bioinformatic analysis of clinical information derived from The Cancer Genome Atlas (TCGA) database, we found that the ferroptosis‐related genes *CHAC1* *,* *NOX4* and *HIF1A* might be the valid indicators for predicting the outcomes of STAD patients. To confirm our speculation, we used R scripts and website tools to conduct several bioinformatics analyses to investigate the clinicobiological function of *CHAC1*, *NOX4* and *HIF1A* and the therapeutic potential of *CHAC1*, *NOX4* and *HIF1A* in stomach cancer. Our speculation was verified in clinical specimens through immunohistochemistry (IHC). Based on the above findings, we conclude that a ferroptosis‐related prognostic model constructed by *CHAC1*, *NOX4* and *HIF1A* might be a reliable prognostic signature for STAD patients.

## 2 METHODS

### 2.1 Data acquisition and processing

The RNA‐seq profile and clinical information of 300 STAD samples, and 30 normal samples were downloaded from TCGA website (https://portal.gdc.cancer.gov/) on 23 April 2021. Then, the GTF annotation file was used to convert the Ensembl gene ID to the gene symbol. This TCGA‐STAD cohort was set as the training group for this study. The GEO dataset GSE15459, comprised of genome‐wide mRNA expression profiles of 192 primary stomach adenocarcinoma (STAD) tissues, was the validation set in our study. A total of 144 ferroptosis‐related genes were retrieved from the manually curated database as possible regulators of ferroptosis FerrDb (http://www.zhounan.org/ferrdb).

### 2.2 Identification of significantly different genes

The ‘LIMMA’ R package was used to identify significantly different genes (SDGs) by the Wilcoxon test. The cut‐off values were determined according to the parameters *p* < 0.01 and false discovery rate <0.01.

### 2.3 Functional enrichment analysis

The ‘clusterProfiler’ R package was used to analyse the functional enrichment of significantly differentially expressed genes, including biological process (BP), cellular component (CC), molecular function (MF) of the Gene Ontology (GO) and 8 kinds of biological metabolic pathways of the Kyoto Encyclopedia of Genes and Genomes (KEGG) analysis.

### 2.4 Construction and verification of the ferroptosis‐related prognostic model

Ferroptosis‐related genes that were highly correlated with the prognosis of STAD were screened by univariate Cox regression analysis and a least absolute shrinkage and selection operator (LASSO) Cox regression model. Then, each STAD sample risk score was calculated by the following formula:

$$\text{Risk}\;\text{score}\;=\;\sum_{i=1}^{n}{\text{Coef}}_{i}\;\times \;X_{i}$$

Coef represents the gene coefficient and X reflects the gene expression level. We divided the samples into high‐ and low‐risk groups bounded by the median of the risk score.

Kaplan‐Meier survival analysis and time‐dependent ROC curve analysis were applied to evaluate the prognostic capability of the signature. We evaluated whether the clinical characteristics and risk scores were risk factors for the prognosis of STAD by univariate Cox regression analyses and calculated the hazard ratio with the ‘survival’ R package. Then, the same procedures were applied to the GSE15459 data to verify the reliability of the prognostic model.

Furthermore, we designed a nomogram to estimate the 1‐, 3‐ and 5‐year survival probability, and the risk score was used as one of the prognostic factors. To compare the calculated rates with the probabilities predicted by the nomogram, a calibration curve describing the 3‐year overall survival (OS) was plotted. The nomogram and calibration curves were constructed by the ‘rms’ R package.

### 2.5 Verification of the clinical samples

Fifty‐one pairs of archived fresh frozen tumour specimens from STAD patients who underwent surgery from August 2016 to September 2017 in our centre were included in this study as another validation set. Basic clinical information was collected from all enrolled patients, including age, sex and tumour staging (according to the eighth edition of the American Joint Committee on Cancer (AJCC) TNM staging system for gastric cancer). These clinical characteristics are shown in Table 1. All aspects of this study were approved by the ethics committee of the Qingdao University School of Medicine. Informed consent was obtained from each participant.

**TABLE 1:** Basic characteristics of STAD patients

Characteristic	*N* = 51	Frequency (%)
Sex		
Male	41	80.4
Female	10	19.6
Age		
≥60	31	60.8
<60	20	39.2
Tumour size		
T1	3	5.9
T2	1	2.0
T3	33	64.7
T4	10	19.6
Unknown	4	7.8
Lymph nodes		
N0	6	11.8
N1	5	9.8
N2	14	27.5
N3	20	39.2
Unknown	6	11.8
Metastasis		
Yes	5	9.8
No	46	90.2
Stage at diagnosis		
I	3	5.9
II	7	13.7
III	30	58.8
IV	5	9.8
Unknown	6	11.8

Fifty‐one freshly frozen tumour specimens from archived STAD patients were immunohistochemically stained (TMA). The expression patterns and levels of *CHAC1 NOX4* and *HIF1A* were determined using IHC assays according to the manufacturer’s instructions (Cell Signaling Technology). In brief, the paraffin‐embedded slides were baked for 1 h at 68°C before xylene deparaffinization and subsequent rehydration through graded ethanol (100% and 95%). Antigen retrieval was performed by boiling the sections in citrate buffer (pH 6.0) for 3 min followed by cooling and then washing with PBS three times. Each section was incubated overnight at 4°C with the following primary antibodies at the indicated dilutions: *NOX4* (14347‐1‐AP), 1:200; *CHAC1* (15207‐1‐AP), 1:200; *HIF1A* (20960‐1‐AP), 1:50 (all from Proteintech Group). Following this, the slide was blocked for 1 h with normal goat serum, incubated with the secondary antibody at 37°C for 30 min, rinsed with TBS for 3 min, counterstained with Harris hematoxylin for 15 s, dehydrated and mounted on coverslips.

All sections were scanned on NanoZoomer slide scanners (NanoZoomer‐XR C12000, Hamamatsu) and viewed with NDP.view software (NDP.view2 U12388–01, Hamamatsu). The MOD (Mean of IOD) of each section was calculated according to the average ODs of the five views by Image‐Pro Plus 6.0 (Media Cybernetics, Inc.). Fragments per kilobase of transcript per million fragments mapped (FPKM) were used to calculate the RNA expression levels in tissues derived from the TCGA database.

### 2.6 Evaluation of the tumour microenvironment (TME) and infiltrated immune cells

To estimate the amount of stromal and immune cells in the tumour tissues, expression data (ESTIMATE) analysis was conducted to calculate the stromal score, immune score, ESTIMATE score and tumour purity of each tumour sample.^8^, ^9^ The Cibersort^10^ was applied to assess different TMEs and infiltrated immune cells in the high‐ and low‐risk groups. Based on a leukocyte gene signature matrix, termed LM22, Cibersort can provide an estimation of the abundances of 22 kinds of immune‐related cells in a mixed cell population. The 22 cell types include naive and memory B cells, resting and activated dendritic cells (DCs), eosinophils, three macrophage types (M0, M1 and M2), resting and activated mast cells, monocytes, neutrophils, resting and activated natural killer cells, plasma cells and seven T‐cell types (CD8, naive CD4, resting memory CD4, activated memory CD4, follicular helper, regulatory Tregs and gamma delta).

### 2.7 Statistical analysis

In this study, we used Strawberry Perl for Windows (Version 5.18.2) to organize the data and used Student’s *t* test to screen for differentially expressed genes between tumour and normal tissues. Pearson’s correlation analysis was applied to compare the correlations between two sets of data and to calculate the correlation coefficient. R (4.1.0) was applied for all statistical analyses and graphing, and *p* < 0.05 was considered statistically significant.

## 3. RESULTS

3

### 3.1 Differential expression of ferroptosis‐related genes and functional enrichment analysis

**FIGURE 1:**
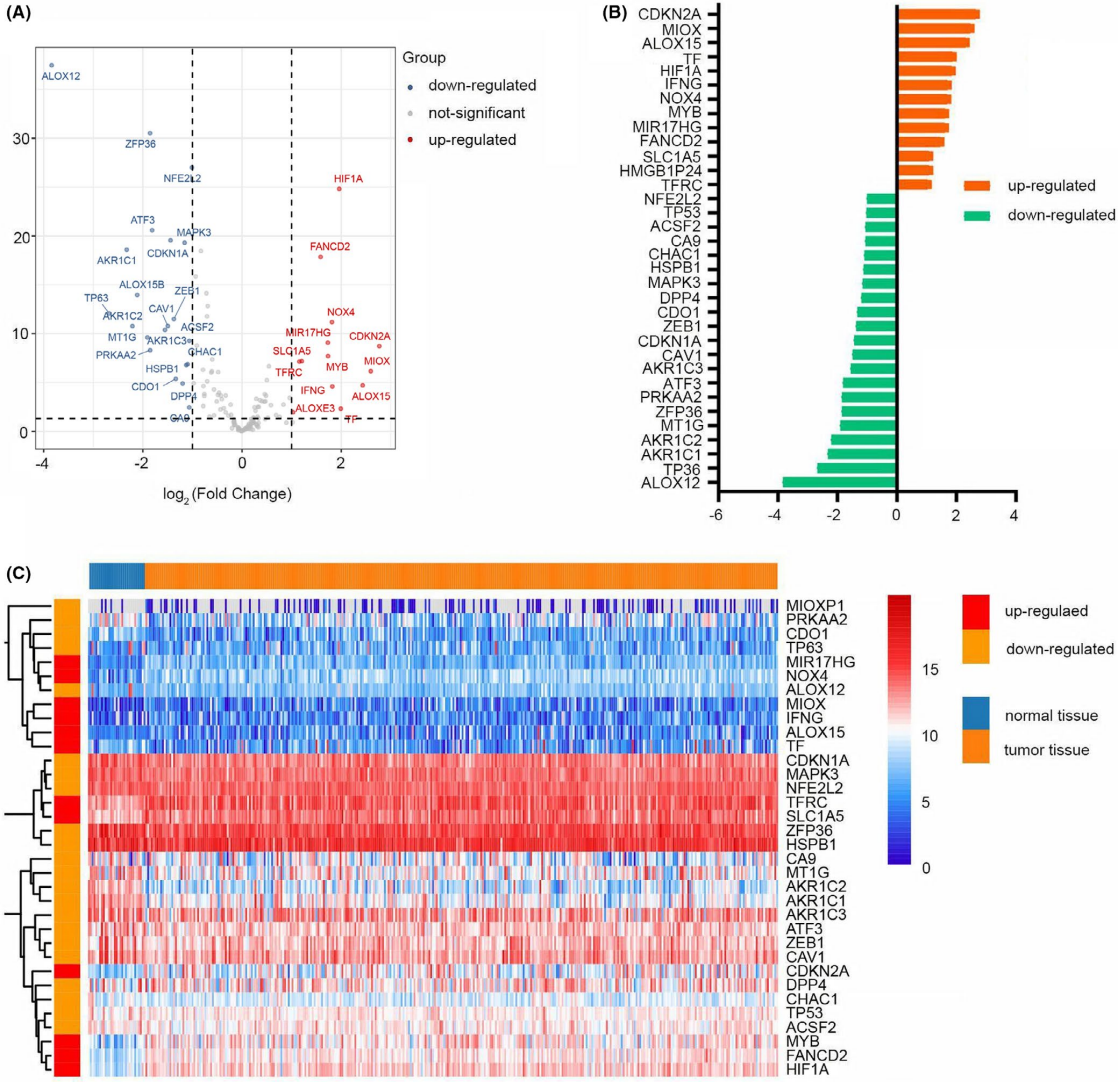
SDG in normal and STAD tissues. (A) Volcano plot of SDG. Red dots represent upregulated genes in STAD tissues, blue dots represent downregulated genes and grey dots represent genes with no significant differences. (B) Deviation plot of SDG. Orange bars represent 13 upregulated genes; green bars represent 21 downregulated genes. (C) Heatmap of the SDG to visualize the expression levels of the genes

RNA‐seq data of ferroptosis‐related genes were extracted from 300 STAD samples and 30 normal samples in the TCGA database. Then, we used the ComBat method to remove batch effects by the R package ‘sva’. Univariate Cox regression analysis showed that between normal controls and STAD patients, 34 out of 144 ferroptosis‐related genes were significantly differentially expressed (*p* < 0.05; Figure 1A). Thirteen of these genes were upregulated, while the other 21 genes were downregulated in the STAD samples (Figure 1B,C).

**FIGURE 2:**
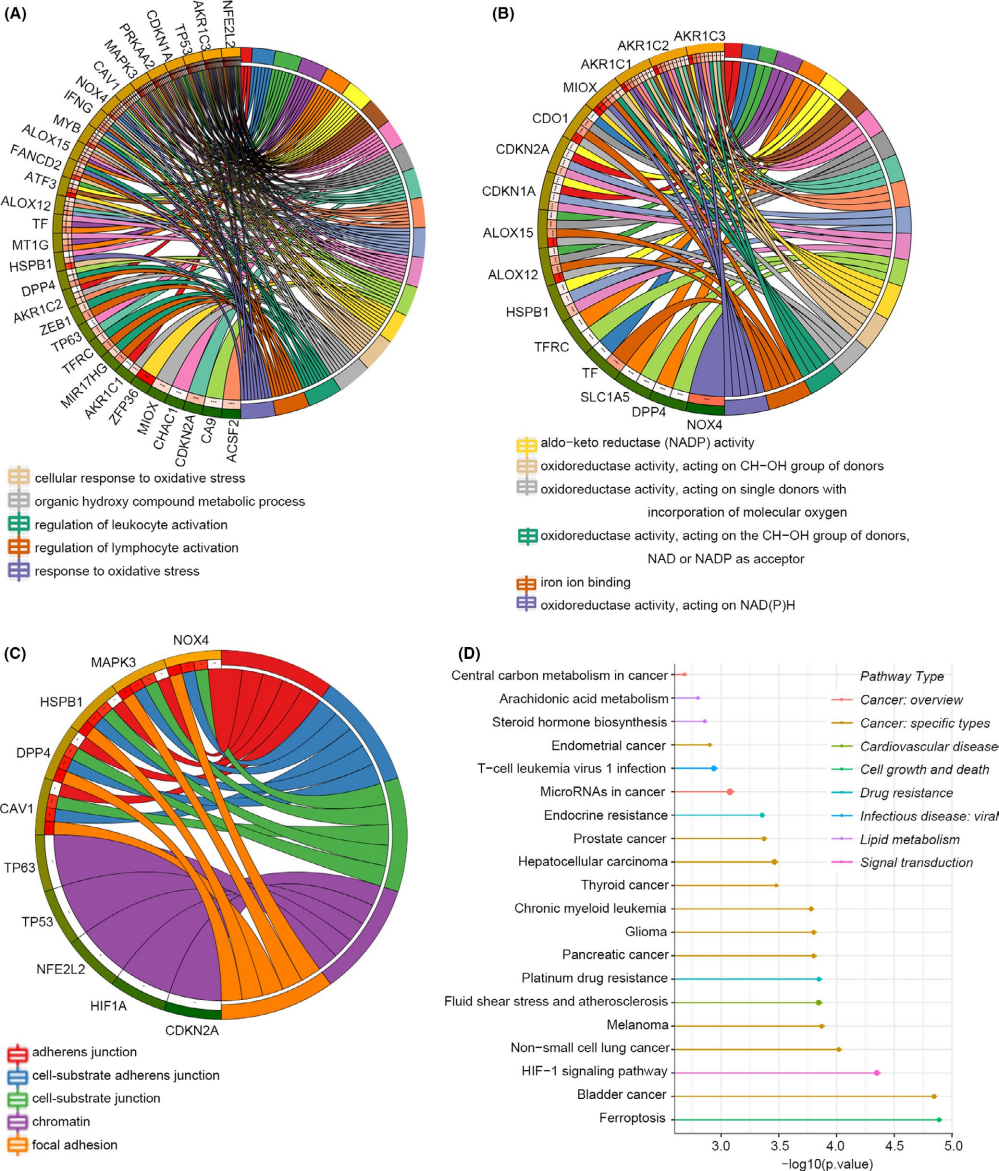
Functional enrichment analysis of SDG. (A–C) Biological process, cellular component and molecular function of GO analysis. (D) KEGG pathways. A larger circle indicates that more genes are enriched

We further investigated the 34 genes’ corresponding biological functions and pathways in ferroptosis by GO and KEGG functional enrichment analysis (Figure 2A–D). The GO results show that these genes are strongly enriched in oxidative stress response and inflammatory response, including response to oxidative stress (BP), regulation of leukocyte and lymphocyte activation (BP) and oxidoreductase activity acting on NADPH (MF). The KEGG results showed that the ferroptosis, HIF‐1 signalling and platinum drug resistance pathways were significantly enriched.

### 3.2 Establishment and verification of a ferroptosis‐related prognostic model in STAD

**FIGURE 3:**
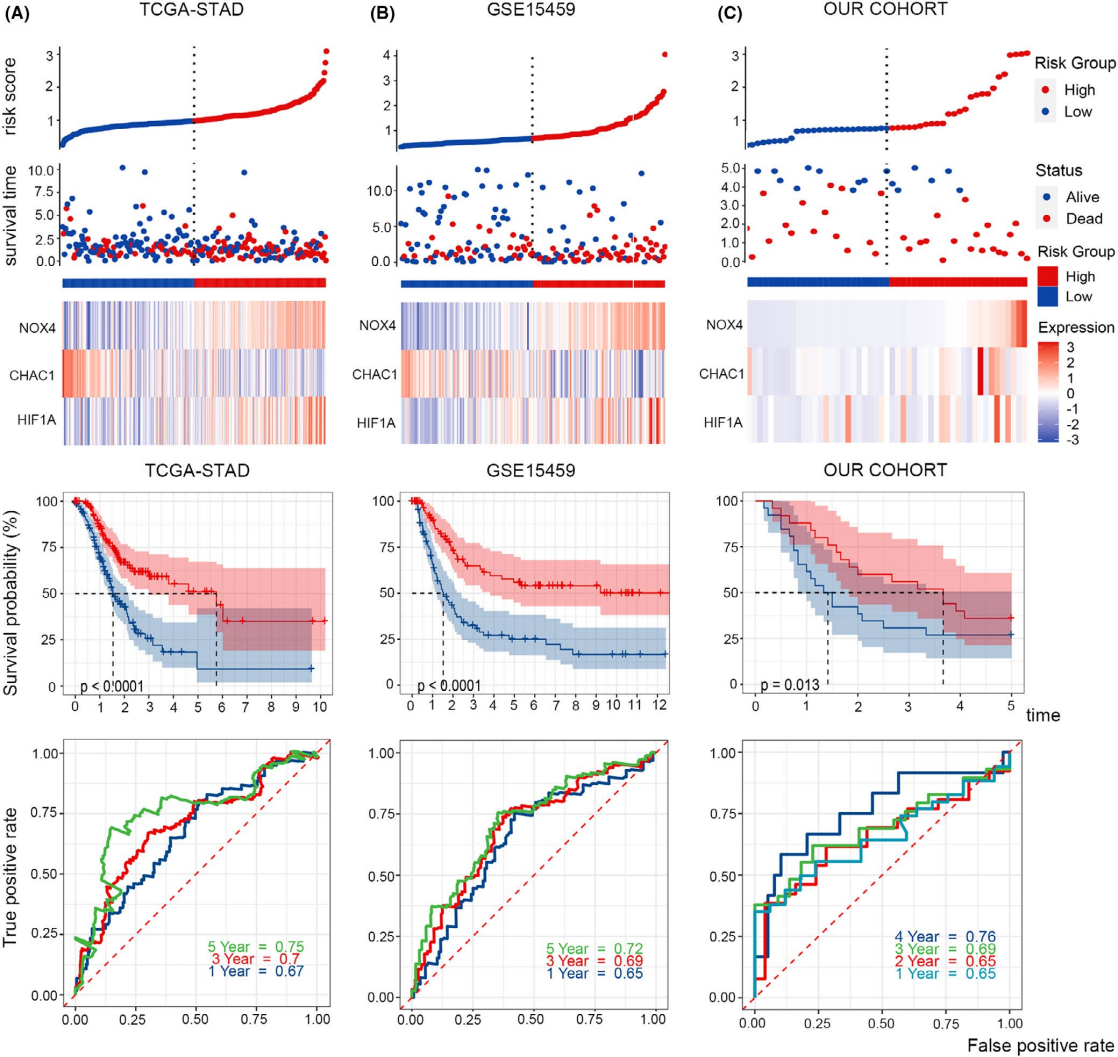
Survival and ROC analysis. Risk score distribution, survival overview, heatmap of key genes, Kaplan‐Meier overall survival curves and time‐dependent ROC curves in TCGA (A), GSE15459 (B), and our cohort (C). As the risk score increased, more patients died

We eliminated 14 patients who were lost to follow‐up. According to the RNA‐seq profile of 144 ferroptosis‐related genes from 286 tumour samples and the corresponding clinical information in TCGA‐STAD, univariate Cox regression analysis showed that 13 genes were significantly associated with the OS of STAD. Then, lasso regression identified three out of the 13 ferroptosis‐related genes that were the most powerful prognostic markers. Finally, multivariate Cox regression was conducted to establish a prognostic model based on the three genes. The formula of the ferroptosis risk score was as follows: risk score = (−0.176 × *CHAC1* expression level) + (0.134 × *NOX4* expression level) + (0.139 × *HIF1A* expression level). The STAD patients were divided into high‐ and low‐risk groups according to the median risk score of 1.012. The distribution of the risk scores, survival times and expression levels of the three genes in the different groups is presented in Figure 3A. The results show that in the high‐risk group, death states are denser, *NOX4* and *HIF1A* are highly expressed, and *CHAC1* expression is reduced.

Kaplan‐Meier survival curves showed that the survival probability of STAD patients with a high‐risk score was significantly lower than that of patients with a low‐risk score. To evaluate the efficiency of the prognostic model, we created a receiver operating characteristic curve (ROC). The area under the curve (AUCs) for 1, 3 and 5 years was 0.67, 0.7 and 0.75 respectively (Figure 3A).

Furthermore, 180 samples from GEO: GSE15459 and 51 samples from our own cohort were used to validate the reliability and accuracy of the model (Figure 3B,C). Twelve patients in GSE15459 who were lost to follow‐up were eliminated. The Kaplan‐Meier survival curves of the two test cohorts both showed that the survival probability in the high‐risk group was significantly shorter than that in the low‐risk group (*p* < 0.0001). The areas under the curve (AUCs) of the ferroptosis risk score in the GSE15459 set for 1, 3 and 5 years were 0.65, 0.69 and 0.72, and the AUCs in our cohort set for 1, 2, 3 and 4 years were 0.65, 0.65, 0.69 and 0.76. These results demonstrated that this model was a powerful predictor of STAD patient outcomes.

**FIGURE 4:**
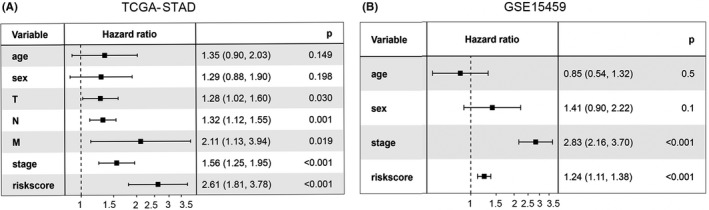
Forest plot. Univariate Cox regression analyses suggested that tumour stage and risk score were independent prognostic factors in (A) TCGA and (B) GSE15459

We then applied univariate Cox regression analysis to verify whether the ferroptosis‐related prognostic signature was an independent prognostic factor for patients with STAD. The tumour staging and ferroptosis‐related prognostic signature were significantly correlated with OS (*p* < 0.001; Figure 4A,B).

### 3.3 Construction of the nomogram

**FIGURE 5:**
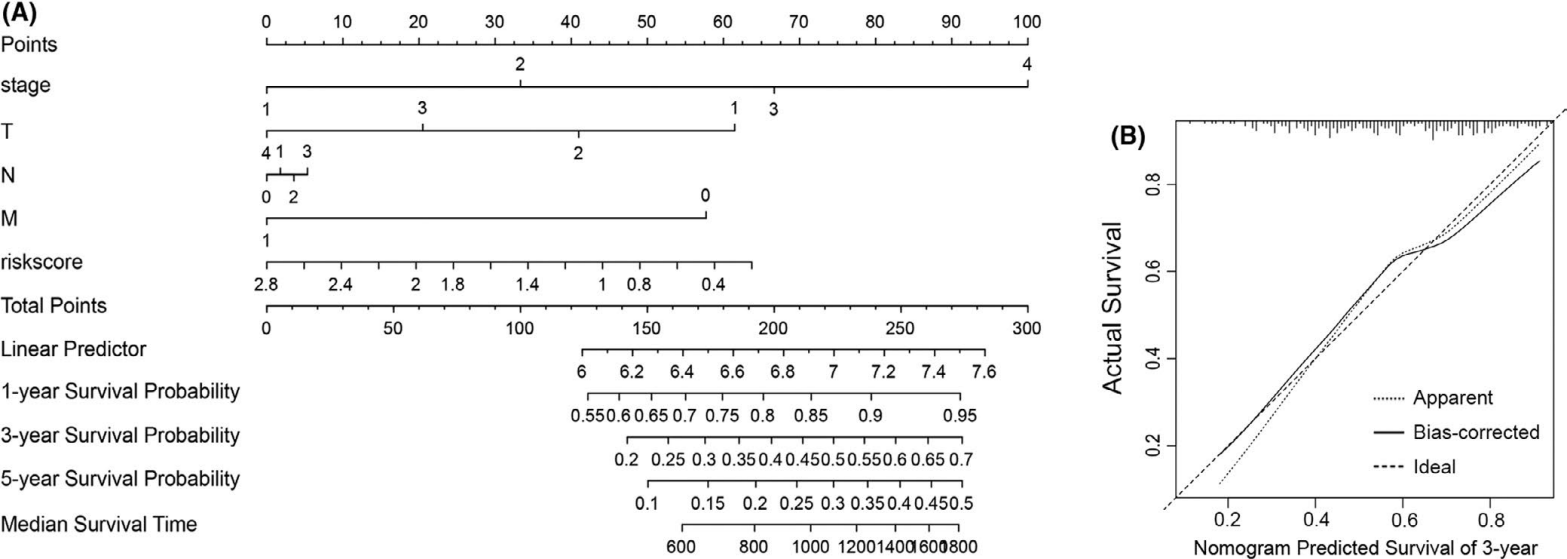
Nomogram and calibration curve. (A) Based on each patient's variable value, draw an upward vertical line to the ‘Points’ bar to calculate points. Then, all the values are summed, and a downward vertical line is drawn from the ‘Total Points’ line to calculate the 1‐, 3‐ and 5‐year survival probability and the median survival time. (B) Calibration curve for the probability of 3‐year survival

Nomograms are commonly used to intuitively evaluate patient prognosis in oncology.^11^ Here, we constructed a nomogram to graphically depict a statistical prognostic model that generates a probability of cancer death for a given individual with STAD. To estimate the survival probability of the STAD patients, we integrated some clinicopathological factors, including age, sex, stage, and T, N and M stages, as well as the prognostic characteristics of the ferroptosis‐related genes to construct a nomogram (Figure 5A). It can be applied to predict the 1‐, 2‐ and 3‐year survival probability. Additionally, the calibration curve showed that the 3‐year actual survival was highly consistent with the predicted values (Figure 5B), indicating that the nomograph is reliable and accurate. This may be helpful for clinicians to make decisions and personalize treatment for STAD patients.

### 3.4 Assessment of the immune microenvironment in STAD

**FIGURE 6:**
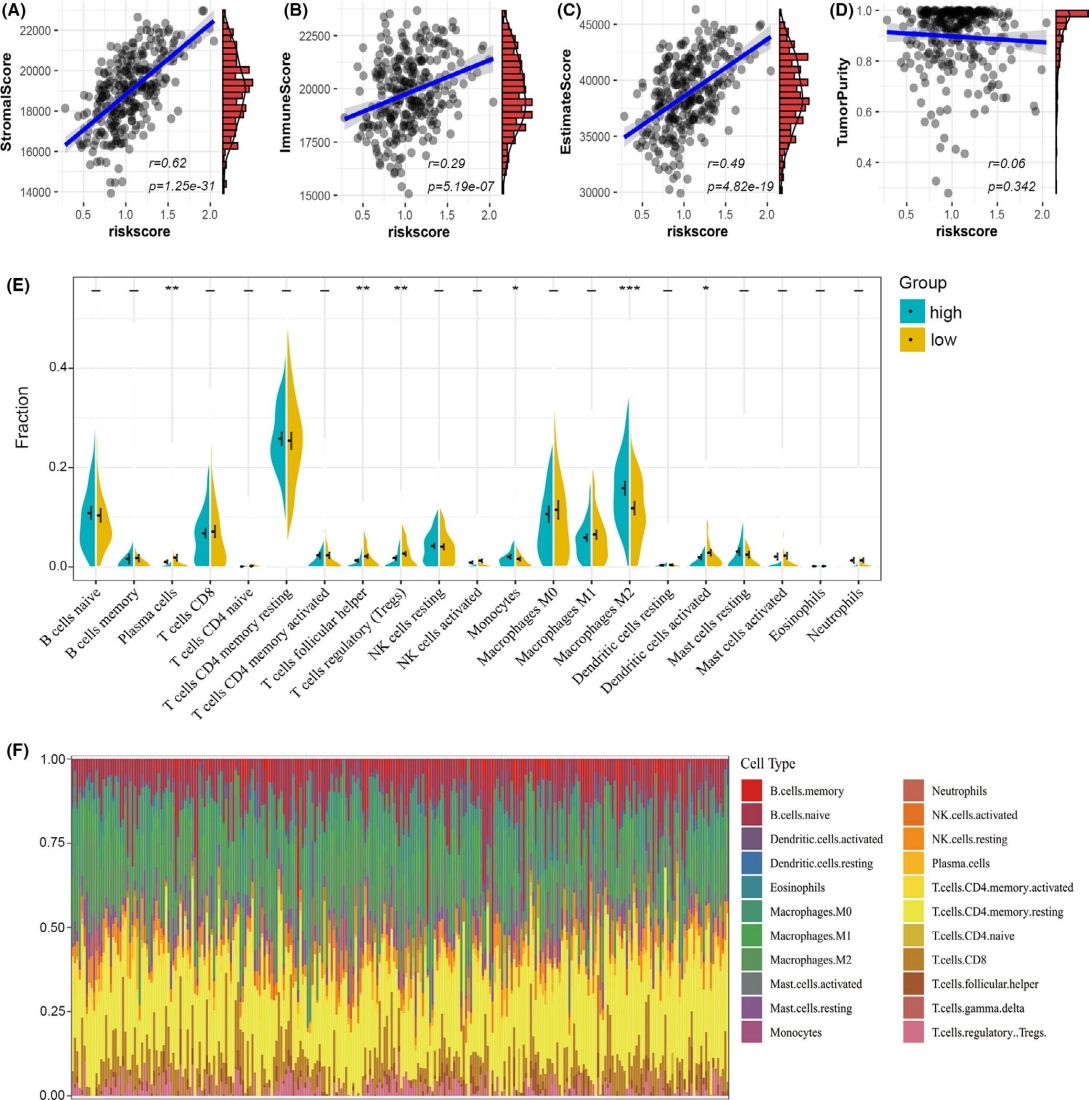
TME cell infiltration in two clusters. (A–D) The correlation between the risk score and stromal score (A), immune score (B), ESTIMATE score (C) and tumour purity (D) in TCGA. (E) The violin plot reveals the abundance of 22 infiltrated cell types in two clusters by the CIBERSORT algorithm. (F) The bar plot shows the proportions of the 22 infiltrated immune cells

We further investigated the correlation between the ferroptosis‐related prognostic signature and immune infiltration in STAD. By conducting ESTIMATE analysis, we found that the risk score of the ferroptosis‐related prognostic signature was significantly positively correlated with the stromal score, immune score and ESTIMATE score (*p* < 0.05) (Figure 6A–C). In addition, there was no obvious correlation between tumour purity and the risk score (Figure 6D).

Then, we used a deconvolution algorithm based on support vector regression and Cibersort immune analysis to determine the type and composition proportion of 22 types of immune cells in the tumours (Figure 6F). We also compared the component differences in immune cells between the two groups (Figure 6E). The results showed that the plasma cells, follicular helper (TFH) T cells, regulatory T cells (Tregs), monocytes, M2 macrophages and DCs activated in the high‐risk group were significantly different from those in the low‐risk group. In addition to monocytes and M2 macrophages, the infiltration of other macrophages was significantly lower in the high‐score group than in the low‐score group. These results suggested that the ferroptosis‐related prognostic signature was associated with immune infiltration.

### 3.5 *CHACI* and *NOX4* were differentially expressed in our human STAD cases

**FIGURE 7:**
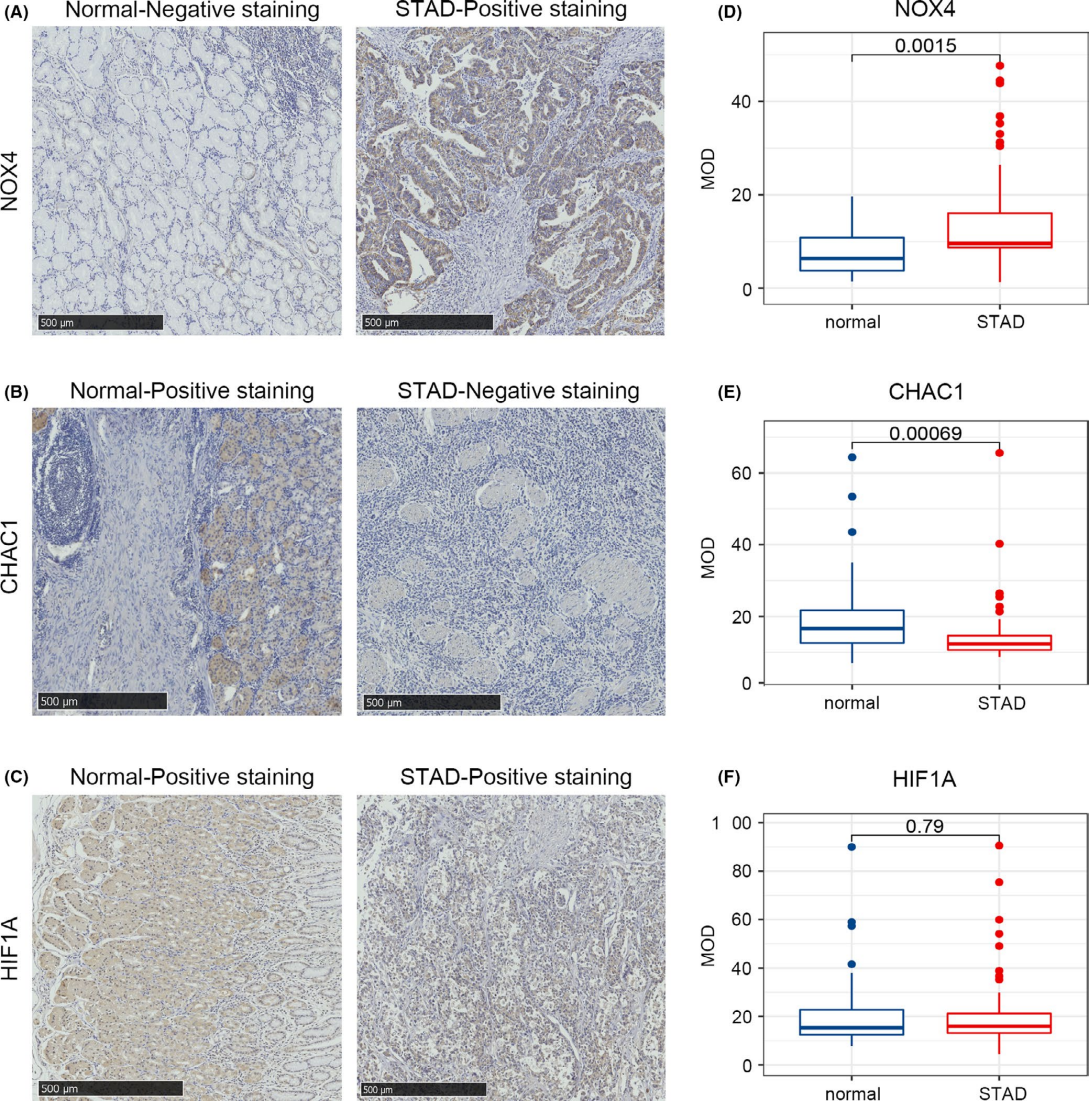
Verification of differential expression of the key genes. (A–C) Representative immunohistochemistry images of STAD tissue and the corresponding paracarcinoma tissues of *CHAC1* (A), *NOX4* (B) and *HIF1A* (C). (D–F) Differences in the protein expression of the key genes in STAD tissues and normal tissues in the clinical samples by immunohistochemistry

To further support our findings, we evaluated *CHAC1*, *NOX4* and *HIF1A* expression in 51 pairs of STAD tissues and the corresponding paracarcinoma tissues (Table S1, Figure 7A–C). Then, we applied Image‐Pro Plus 6.0 to estimate the expression levels of the three genes in each sample and plotted a boxplot to intuitively show the expression differences (Figure 7D–F). We found that the proportion of samples with high expression of *NOX4* in STAD tissues was significantly higher than that in paracancerous tissues, while *CHAC1* showed the opposite trend. However, there was no significant difference in *HIF1A* expression between the two groups, this may be due to a bias in sample distribution. Next, the estimated expression level was substituted into the prognostic model to verify the reliability of this signature.

## 4 DISCUSSION

Ferroptosis, driven by unrestricted lipid peroxidation, is a newly defined form of regulated cell death.^12^ It is involved in various pathophysiological conditions of several diseases, such as metabolic disease^13^ and various neurodegenerative diseases.^14^ Recent preclinical studies have revealed that ferroptosis may be an attractive therapeutic target in pancreatic cancer,^15^ lung cancer and liver carcinoma.^16^ Therefore, we investigated the significance of ferroptosis‐related genes in STAD, aiming to find new biomarkers for STAD therapy.

Among the 144 ferroptosis‐related genes, 34 were significantly different between STAD and normal samples from the TCGA‐STAD dataset. GO analysis of these 34 genes indicated that oxidative stress and the inflammatory response were significantly enriched. This result is consistent with Chen's^17^ conclusion that the ferroptotic response can be affected by various oxidative stress and antioxidant defense pathways. Various inflammatory mediators are produced by arachidonic acid metabolism in ferroptotic tissues.^18^ The KEGG results found that the HIF‐1 signalling pathway and platinum drug resistance were significantly enriched, which can provide directions for STAD targeted therapy and drug resistance.

A prognostic model was constructed based on three ferroptosis‐related genes, *CHAC1*, *NOX4* and *HIF1A*. ROC curves, calibration curves and forest plots verified that the constructed prognostic signature could accurately predict OS. The *CHAC1* enzyme is involved in the γ‐glutamyl cycle and can degrade glutathione. Increased expression of *CHAC1* can lead to glutathione depletion and an imbalance in cellular REDOX levels.^19^ Tomohisa et al.^20^ found that overexpression of *CHAC1* may contribute to the development of stomach cancer in *Helicobacter pylori*‐infected parietal cells. NADPH oxidases (nicotinamide adenine dinucleotide phosphate oxidase, NOXs) are the major source of ROS in cancer cells.^21^ *NOX4* can be induced upon hypoxia or ischemia.^22^ Multiple studies have found that the upregulation of *NOX4* can promote the occurrence and metastasis of tumours.^23^, ^24^ *HIF1A* is one of the main regulators of tumour cell adaptation to the hypoxic microenvironment. When cells are deprived of oxygen, *HIF1A* expression is upregulated, which activates some oncogenes.^25^ A recent study has shown that *HIF1A* signalling leads to a significant increase in *NOX4* expression.^26^ Thus, the three ferroptosis‐related genes in the prognostic signature have been validated to be involved in tumorigenesis and progression in various studies. In addition, both the univariate Cox regression analysis of the risk score and the calibration curve of the nomogram verified the reliability of this model. The established prognostic model can help determine the prognosis of patients with STAD.

Subsequently, by applying the ESTIMATE and Cibersort algorithms, we studied the TME’s immune cell infiltration. We found that M2 macrophages, which promote tumour growth and invasion,^27^ were significantly increased in the high‐risk group. A recent study demonstrated that follicular helper T cells, which were significantly increased in the low‐dose group, exerted antitumour effects in a CD8^+^‐dependent manner. Moreover, the presence of TFH is essential for the efficacy of PD‐1/PD‐L1 therapy.^28^ However, Tregs that can prevent any overt immune response^29^ were highly expressed at low levels. The estimation analysis indicated that the degree of immune cell infiltration was significantly positively correlated with the risk score. This result may be related to the large proportion of tumour‐associated macrophages (TAMs) in the TME. By regulating tumour cell metabolism, TAMs can promote tumour growth.^30^ Thus, the risk score calculated by the ferroptosis‐related prognostic model can represent the TME of STAD patients to a certain extent.

## 5 CONCLUSION

In conclusion, our study describes a ferroptosis‐related gene‐based prognostic model for STAD that is significantly correlated with OS, clinical characteristics, TMB and the tumour microenvironment. Moreover, the reliability of the prognostic model and the expression differences of related genes in STAD were verified through our clinical samples. However, this study was performed at a machine learning level, and further in vivo and in vitro experiments are needed to validate the findings.

## CONFLICT OF INTEREST

The authors declare that the research was conducted in the absence of any commercial or financial relationships that could be construed as a potential conflict of interest.

## AUTHOR CONTRIBUTIONS

**Ruoxi Xiao:** Conceptualization (equal); writing – original draft (lead). **Shasha Wang:** Formal analysis (equal); writing – original draft (equal). **Shufen Zhao:** Project administration (equal); writing – review and editing (equal). **Jing Guo:** Project administration (equal); writing – review and editing (lead). **Shihai Liu:** Formal analysis (equal); writing – review and editing (equal). **Aiping Ding:** Project administration (equal); writing – review and editing (equal). **Gongjun Wang:** Methodology (equal); resources (lead). **Wenqian Li:** Methodology (equal); validation (equal). **Yuqi Zhang:** Formal analysis (equal); validation (equal). **Xiaoqian Bian:** Methodology (equal); visualization (equal). **Wensheng Qiu:** Project administration (equal); writing – review and editing (equal).

## Supporting information

Table S1

## Data Availability

The data that support the findings of this study are available from the corresponding author upon reasonable request.
